# Global seasonal prediction of fire danger

**DOI:** 10.1038/s41597-024-02948-3

**Published:** 2024-01-25

**Authors:** Francesca Di Giuseppe, Claudia Vitolo, Christopher Barnard, Giorgio Libertá, Pedro Maciel, Jesus San-Miguel-Ayanz, Sebastien Villaume, Fredrik Wetterhall

**Affiliations:** 1 Europen Centre for Medium-Range Weather Forecast, Reading (ECMWF), Reading, United Kingdom; 2 European Space Agency (ESA), Frascati, Italy; 3 Joint Research Center (JRC), Ispra, Italy

**Keywords:** Natural hazards, Environmental sciences

## Abstract

The European Centre for Medium range weather forecast (ECMWF) on behalf of the Copernicus Emergency Management Service (CEMS) has recently widened the fire danger data offering in the Climate Data Store (CDS) to include a set of fire danger forecasts with lead times up to 7 months. The dataset incorporates fire danger indices for three different models developed in Canada, United States and Australia. The indices are calculated using ECMWF Seasonal Forecasting System 5 (SEAS5) and verified against the relevant reanalysis of fire danger based on the ECMWF Re-Analysis (ERA5). The data set is made openly available for the period 1981 to 2023 and will be updated regularly providing a resource to assess the predictability of fire weather at the seasonal time scale. The data set complements the availability of seasonal forecast provided by the Copernicus Emergency Management Service in real time.

A preliminary analysis shows that globally anomalous conditions for fire weather can be predicted with confidence 1 month ahead. In some regions the prediction can extend to 2 months ahead. In most situations beyond this horizon, forecasts do not show more skill than climatology. However an extended predictability window, up to 6-7 months ahead is possible when anomalous fire weather is the results of large scale phenomena such as the El Niño Southern Oscillation and the Indian Ocean Dipole, often conducive of extensive fire burning in regions such as Indonesia and Australia.

## Background & Summary

Wildfires are processes that can be both beneficial and deleterious for the environment. On the one hand, uncontrolled fires make it often in the news as environmental disasters, causing destruction and loss of lives. On the other hand, fires have been happening since hundreds of million years ago (according to tests on fossil charcoal^1^) and have a crucial role in the evolutionary path of many ecosystems^2^. In addition, controlled fires are very efficient for clearing agricultural land and for fire prevention and management, e.g. controlled burns create a discontinuity in the land depriving fires from fuel and interrupting potential propagation pathways^3^. Hence the importance of managing wildfires and prevent as much as possible that controlled and accidental burns rage out of control. Forecasting fire danger is key in fire prevention and protection measures as it improves readiness of fire professionals and allows timely and efficient allocation of resources^4^.

Scientific literature shows that, besides well established fire danger forecasts with lead times of a few days^5,6^, skilful predictions of fire danger is possible up to the seasonal time scale for Mediterranean Europe^7^, United States^8,9^ and Asia^10^. Seasonal forecasting of fire weather conditions throughout the world have been found to correlate with large scale climate patterns such as the El Niño Southern Oscillation (ENSO) and the Indian Ocean Dipole, implying that fire weather conditions can be predicted fairly accurately for various seasons and regions^11^. In Europe, forecasts for the eastern and south-eastern areas have shown to be fairly reliable ‘paving the way to their operational applicability^7^.

The soil moisture and heat wave mechanism has been identified as an important source of predictability in Europe, along with atmospheric circulation patterns such as ENSO^12^ and other atmospheric conditions such as triggering trade-offs between relative humidity and temperature^7,13,14^, although the latter two deserves further investigations.

In 2018, ECMWF in collaboration with the Copernicus Emergency Management Service (CEMS), developed the ECMWF Global Fire Forecasting (GEFF) model^5^ which is run operationally and provides the fire community with pre-calculated fire danger indices based on fire danger indices developed in Canada (Fire Weather Index^15^), United States (U.S. Forest Service National Fire-Danger Rating System^16^, and Australia (McArthur Mark 5 Rating System^17^). Using ECMWF weather forcings, GEFF produces fire danger reanalysis^18,19^ as well as forecast products^5,6^. A set of fire danger seasonal forecast based on ECMWF long range weather prediction system (SEAS5) is available and span the period 1981 to 2023^20^. The dataset will be updated regularly providing an resource to understand the predictability of landscape flammability globally and across several decades. Seasonal forecast have monthly initial date and forecast horizon of 216 days corresponding to 7 months.

This data descriptor reports on the available dataset and makes a first assessment of the skill of the fire danger seasonal prediction using the available fire weather reanalysis data-set as a reference^18,19^. The new dataset could be particularly important to help decision makers and forestry agencies to prepare for periods of potentially high fire activities. It is made available as a probabilistic model output, allowing to quantify uncertainties in the fire danger estimations. The seasonal estimates of fire indices are released under the Copernicus open data license, through the Copernicus Climate Data Store (CDS).

## Methods

Seasonal forecasting aims to offer valuable insights into the anticipated “climate” for the upcoming months. It is important to notice that a seasonal forecast substantially differs from a weather forecast: weather represents momentary and ever-evolving atmospheric conditions, while climate embodies the statistical average of these weather patterns within each season. The primary objective of a seasonal weather forecast is therefore to predict the potential range of mean weather conditions expected in the forthcoming season^21^.

The seasonal weather prediction utilized in constructing the provided database uses a coupled atmosphere-ocean system. Within this system, the atmospheric component is represented by the ECMWF IFS (Integrated Forecast System) model version 43r1^22^, which was initially introduced for medium-range forecasting on November 22, 2016. The seasonal weather forecasts are generated using a horizontal resolution of 0.25 degrees. For the oceanic component, SEAS5 employs the NEMO (Nucleus for European Modelling of the Ocean) community ocean model, configured with a resolution of 0.25 degrees and encompassing 75 vertical levels (known as the ocean model configuration ORCA025z75). To estimate the range of errors in the weather forecast simulations, a 51-member ensemble is generated by perturbing both the initial conditions of the simulation and the model parameters. This ensemble approach aims to provide an estimations of the uncertainties present in seasonal forecasting and its a common methods employed in weather forecast, especially at long lead times^23,24^.

Any coupled model that runs in seasonal forecast mode suffers from bias - the climate of the model forecasts differs to a greater or lesser extent from the observed climate^25^. Since seasonal forecast signals are often small, this bias needs to be considered, and must be estimated from a previous set of forecasts. A set of re-forecasts (otherwise known as hindcasts or back integration or just referred as climatology) are thus made starting on the 1st of every month for the years 1981–2016. They are identical to the real-time forecasts in every way, except that the ensemble size is only 25 rather than 51 and the starting point is a re-analysis instead than an operational analysis. The forecasts generated from this ensemble span 7 months^22^ and is available as a open access dataset through the climate data store^26^. This dataset has found extensive application in operational weather forecasting and validation of its skill has been carried out over the years^27–29^.

When applying the concept of seasonal forecasting to fire danger, the objective is to predict anomalies in landscape flammability, thereby estimating the most probable fire risk for the upcoming season. The provided fire danger indices are computed utilizing the GEFF model using the above described SEAS5 seasonal weather predictions as its driving forcings. The presented dataset maintains an identical structure to its parent weather dataset but encompasses a distinct set of variables specifically tailored to assess the sustainability and intensity of landscape fires once ignited.

There is a non linear relationship between weather anomalies to fire danger anomalies. Therefore, the level of accuracy and predictability observed and validated within SEAS5 forecasts is unlikely to be directly transferable or representative of the same skills observed within the provided dataset. Consequently, this paper introduces an initial, rudimentary validation of the provided dataset. It is evident that numerous unexplored avenues will still exist, including the drivers for fire danger predictability. The primary motivation for making this dataset accessible to the broader scientific community is to encourage and facilitate its further exploration, validation and utilization.

## Data Records

The Global seasonal prediction of fire danger dataset is made available through the Climate Data Store^20^. The CDS offers a comprehensive repository of climatic information, and offers several advantages; open access via a user friendly web interface and bulk access via a convenient API, integration with the CDS toolbox for performing server-side operations as well as shared visualisation and data analysis tools based on notebooks. Users can browse the available data catalogue without logging in, however registering an account is mandatory to download data. The CDS has a user-friendly web interface, ideal for the retrieval of small datasets while for larger data volumes users are encouraged to send data requests using the CDS API.

The fire danger seasonal forecast dataset has a global coverage and a spatial resolution of about 0.25 degrees (about 35 km). Natively, data are laid out over an octahedral reduced Gaussian grid (O320), and archived as GRIB2, a standard format published by the World Meteorological Organisation^30^. Users can also request data in NetCDF format which implies an internal remapping data transformation. Data in NetCDF format are on a regular unprojected grid with spherical coordinates expressed in decimal degrees (EPSG:4326). Latitudes span the range from −90 to +90 degrees and are referenced to the equator. Longitudes are in the range from 0 to 360 degrees, referenced to the Greenwich Prime Meridian, consistently with other ECMWF products. Products from three main fire danger systems are made available:

- The Canadian Fire Weather Index;^15^
- The U.S. Forest Service National Fire-Danger Rating System;^16^
- The Australian McArthur Mark 5 Rating System^17^.

For an in-depth description of the fire rating systems and indices, the reader is reminded to read previous works from Di Giuseppe *et al*^5,6^. as they provide the documentation and analysis of the potential predictability of using short and medium-range weather forecast to derive fire danger. In the subsections below, the three systems are briefly described with the list of the available indices and sub-indices provided (Table 1).

**Table 1:** List of the main variables available in the dataset.

SYSTEM	VARIABLES	DESCRIPTION
FWI	Fine Fuel Moisture Code (FFMC)	Numeric rating representing the moisture content found in fine fuels such as grass, needles, and small twigs
	Duff Moisture Code (DMC)	Numeric rating representing the moisture content of material in the layer of partially decomposed organic matter (known as duff) consisting of leaves, needles, twigs, and other plant debris found on the forest floor,
	Drought Code (DC)	Numeric rating representing the moisture content of deep, compact organic layers within the soil
	Initial Spread Index (ISI)	Fire weather index that estimates the potential rate of fire spread immediately after ignition, taking into account fuel moisture, wind speed, and other weather-related factors.
	Build-up Index (BUI)	Fire weather index used to assess the cumulative effect of moisture deficits in both the duff and litter layers of the forest floor, reflecting the dryness and flammability of deeper organic materials over time
	Fire Weather Index (FWI)	Composite rating used to assess the potential fire behavior. It includes components such as the Initial Spread Index (ISI), Build Up Index (BUI), and Drought Code (DC), providing a comprehensive assessment of fire risk under specific weather conditions.
NFDRS	Burning Index (BI)	Fire weather index that quantifies the potential intensity of a wildfire by considering the energy released per unit length of fire front.
	Ignition Component (IC)	Probability or likelihood of a fire igniting and spreading under specific environmental conditions.
	Energy Release Component (ERC)	Fire weather index used to estimate the potential energy released from a wildfire. It quantifies the total heat output per unit area that a fire might generate
MARK5	Fire Danger Index (FDI)	Fire danger measure that evaluates the potential risk or severity of fires based on various environmental and weather conditions.

### The FWI system

The Canadian FWI system describes the fire weather, the complex atmospheric conditions that can lead to a dangerous fire. It quantifies potential fire danger using temperature, relative humidity, wind speed, and 24-hr accumulated precipitation values measured at noon Local Standard Time (LST). The indices include measures of fuel moisture (Fine Fuel Moisture Code, Duff Moisture Code, and Drought Code), fire behavior indices (Initial Spread Index, and Build Up Index) and indices related to ease of fire suppression (Fire Weather Index and Danger Severity Rating). This is the index used by Environment Canada to assess short range fire danger and also monthly and seasonal fire danger outlooks. The Fire Weather Index (FWI) is widely recognized as the most utilized index globally and has demonstrated its effectiveness across various biomes^6,31,32^. However, its applicability in predicting fire activity in fuel-limited biomes is limited due to the absence of a connection to real time updates on fuel availability^33^.

### The NFDRS system

The National Fire Danger Rating System (NFDRS) is widely used in the U.S. The fire danger is rated accordingly to static maps of fuel type and topography and considers weather as the main driver. It uses temperature, precipitation, relative humidity and cloud cover to estimate the moisture content of dead and live vegetation at different depth in the fuel bed. In turn, these allow to calculate the Ignition Component and contribute to the other indices such as the Spread Component, Energy Release Component and Burning Index. The NFDRS is used by all US federal and state agencies (e.g. The U.S. Department of Agriculture, The National Wildfire Coordination Group, etc.).

### The MARK5 system

The McArthur (MARK5) fire danger rating system is mostly used in Australia. It uses precipitation, temperature, relative humidity and wind speed to estimate the behaviour of fires burning on a typical Australian landscape. At first the Drought Factor is calculate to represent the effect of temperatures and precipitation on fuel drying. The drought factor is then used to calculate the Keetch-Byram Drought Index which measure soil moisture deficit^34^. Lastly, the Fire danger Index, is calculated to quantify probability of fire occurrence, its intensity, and related difficulty of suppression. The McArthur (MARK5) fire danger rating system is mostly used in Australia, by rural fire authorities.

## Technical Validation

In this section, we conduct a validation of the dataset. To the best of our knowledge, publicly available datasets providing seasonal forecasts of fire danger are lacking. Consequently, conducting an inter-comparison is not feasible. Instead, we perform a consistency check to evaluate the dataset’s capability in predicting anomalous conditions at various lead times, comparing it to a reanalysis dataset, which serves as the reference^18,19^.

Although the link between long-term fluctuations of sea surface temperature (SST) and seasonal precipitation/drought patterns are scientifically proven in the tropics and to a lesser extent in the extra-tropics^27,35^, the implications on seasonal fire danger is largely under-explored. As fire danger is, by definition, weather-driven a link with SST is expected to be detectable in terms of long-term averages (typically over one to three month). If this is the case, these forecasts should gain relevance as support to decision-making processes in a wide range of sectors, such as energy, agriculture, water, and risk management^36^. One important aspect is, therefore, whether there is enough forecast skill to assert the usability of seasonal fire danger forecast in real-time applications.

### Global skill

The global skill metrics presented are provided as monthly means and using the ensemble mean as best prediction outcome. Also the FWI is chosen as an example as this is one of the most used metric to predict fire danger in global systems^31,37^. Results are similar for other metrics.

**Fig. 1:**
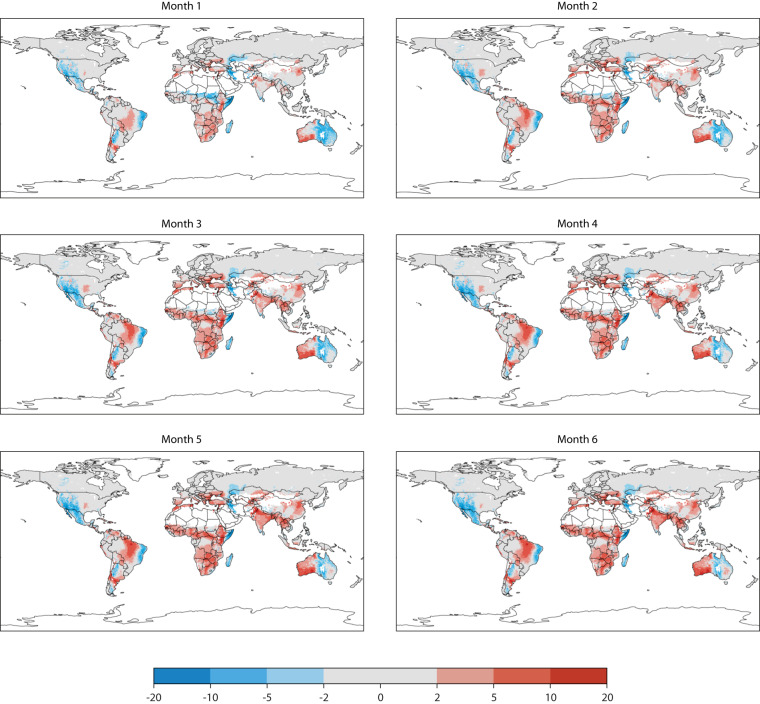
Bias as the average deviation or difference between the predicted monthly values and the observed values here provided by reanalysis simulations. It provides information about the tendency of the model to consistently overestimate or underestimate the true values. The average is performed for the ensemble mean and for all the months in the 1981–2020 period. Panel a to f provides the 7 months forecast horizon available.

**Fig. 2:**
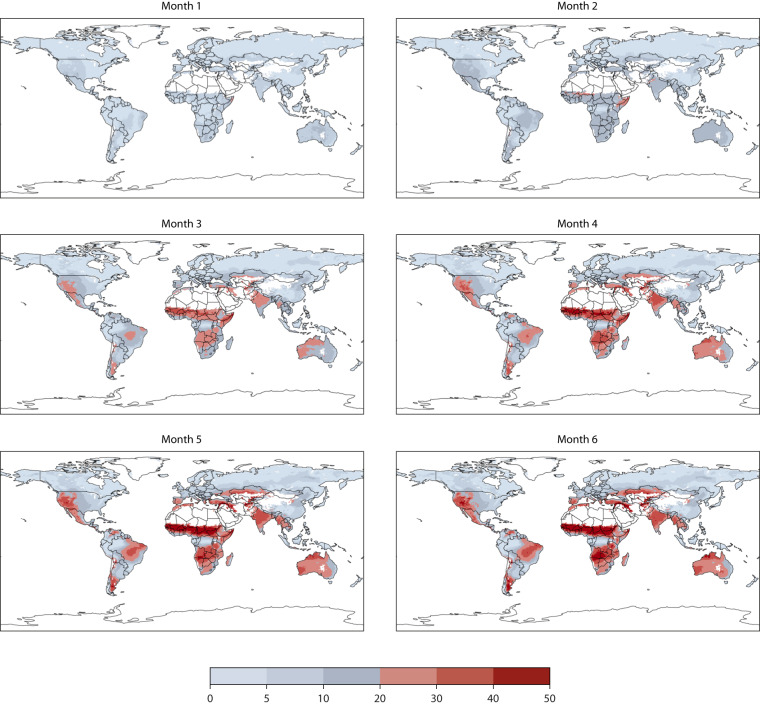
Same as Fig. 1 but for the RMSE.

Both bias and root mean square error are used for assessing model performance (Figs. 1 and 2), as they capture different aspects. Bias helps identify consistent deviations from the true values, while RMSE provides an overall measure of accuracy, considering both bias and the spread of errors. They provide insights into the systematic errors and overall quality of the model’s predictions compared to the reference value identified as ERA5 fire danger reanalysis^19^. A positive bias indicates an over-prediction the opposite for negative bias. Biases tend to increase for more distant prediction while they have similar spatial distribution as they typically diagnose the systematic deficiency of the underlying weather forecast model.

Regional differences in forecasting FWI compared to ERA5-derived databases can be attributed to forecast skills, which significantly impact FWI simulation accuracy. Although temperature prediction skills exhibit global uniformity, forecasting precipitation across various global models, including the ECMWF model, presents a complex scenario. Notably, mid-latitude precipitation forecasting demonstrates higher accuracy due to its linkage with frontal systems driven by large-scale dynamics^38^. Conversely, convective precipitation, predominant in the tropics, poses a challenge due to its stochastic nature. Over time, advancements have narrowed the gap, yet forecasting accuracy remains inferior in the southern extra-tropical region compared to the Northern Hemisphere due to better initial forecast constraints stemming from an enhanced observing system^28^. These insights largely account for the superior FWI prediction performances in the Northern Hemisphere when averaged during the years. Notably, forecasting skills exhibit significant year-to-year variations, particularly during occurrences of large-scale phenomena such as the El Niño Southern Oscillation (ENSO). These phenomena enhance predictability in both tropic and extra-tropical regions through teleconnections, leading to improved forecasting capabilities^39^.

When the bias and the RMSE are of the same magnitude as the signal of interest, typically in the order of 10 units for the fire weather index, using the prediction is equivalent to employing climatology. It is evident from Figs. 1 and 2 that, on average, after month 2, most of the areas affected by changes in landscape flammability display errors that would render the direct use of fire danger values unsuitable for advance warnings based on warning levels. To extend the usability of seasonal forecast information, the concept of anomalies is often utilized. Model anomalies, i.e., deviations from the model climate, are unbiased with respect to observations and are used to assess deviations from long-term average conditions. They cannot be used at face value but are useful to identify the early establishment of dangerous conditions, which can aid early planning rather than guide suppression actions.

**Fig. 3:**
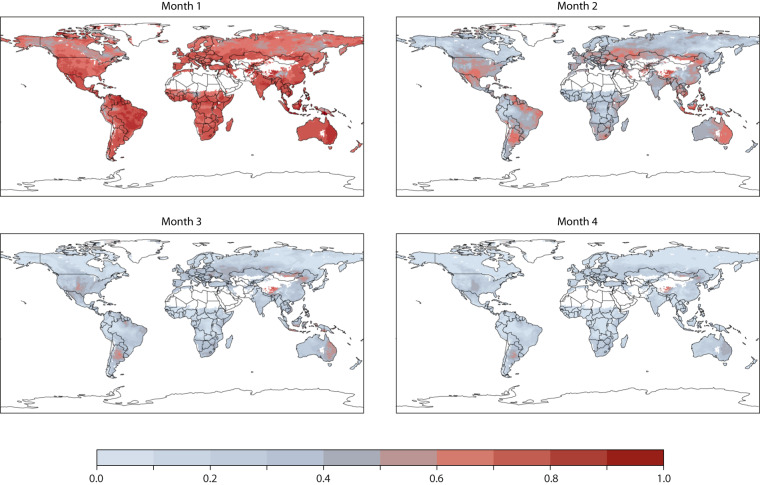
Anomaly correlation for the fwi seasonal forecast system during the hindcast period (1981–2022) for all the the forecasts and valid for month 1 to 4.

The Anomaly Correlation Coefficient (ACC) stands as one of the most widely used measures in verifying spatial fields. It denotes the spatial correlation between a forecast anomaly relative to climatology and the verifying analysis anomaly relative to climatology. ACC serves as a measure of how accurately the forecast anomalies represent the observed anomalies and illustrates the alignment of predicted values from a forecast model with real-life data. ACC values range between +1 and −1. As ACC values approach +1, it indicates substantial agreement, signifying valuable forecast anomaly information. When ACC hovers around 0.5, the forecast errors resemble those of a climatological average-based forecast. An ACC near 0 indicates poor agreement and suggests that the forecast holds minimal value. Figure 3 illustrates the anomaly correlation for the FWI seasonal forecast system throughout the hindcast period (1981–2022) across all forecasts, valid for months 1 to 4. It highlights significant skill in detecting anomalous conditions a month ahead across nearly all regions. In a few areas, anomaly conditions can even be predicted up to 2 months in advance.

## Extended predictability

In this section, we demonstrate how heightened skills in seasonal weather forecasts can enhance predictability in fire danger. We illustrate this by focusing on two regions in the world where large-scale phenomena are known to improve forecast accuracy and analyze their impact on seasonal fire danger forecasts^27^. These results confirm the dataset’s ability to replicate expected predictability patterns. However, conducting a comprehensive global analysis of seasonal forecast skills exceeds the scope of this data descriptor and is deferred to subsequent studies.

### El Niño Soutern Oscillation (ENSO)

El Niño Soutern Oscillation (ENSO) is a climate pattern characterized by the warming of the surface waters in the central and eastern tropical Pacific Ocean and often leads to a shift in rainfall patterns, resulting in reduced precipitation in Southeast Asia, including Indonesia. This can create drier-than-normal conditions, especially in peatland areas, making them more susceptible to fires^40^. The conditions established by strong El Niño conditions exacerbates landscape flammability but are human activities that play a significant role in igniting fires. In Indonesia, particularly in the regions of Sumatra and Kalimantan, land clearing practices such as slash-and-burn agriculture, illegal logging, and peatland drainage for agriculture have been responsible for extensive burning in the past^41^. Release of large amounts of smoke and pollutants into the atmosphere have affected air quality not only within Indonesia but also in neighboring countries, such as Malaysia and Singapore, generating international health emergencies^42,43^.

**Fig. 4:**
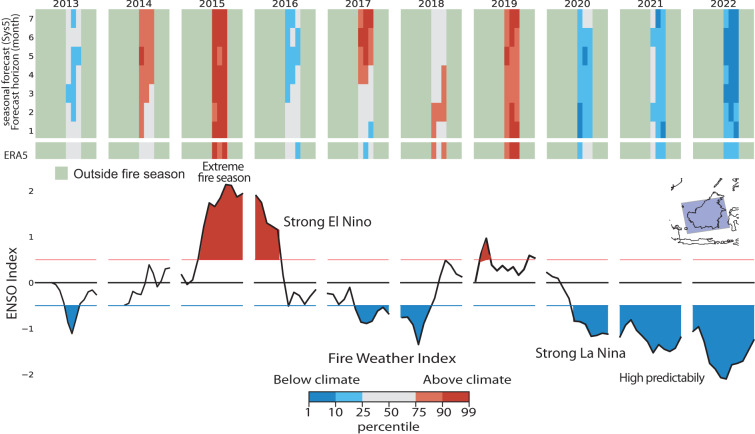
Prediction of monthly means fire danger anomalous conditions between 2013 and 2022 over Indonesia. Months are classified as above or below the 1981–2022 climate mean using percentiles. Anomalies from ERA5 are compared to SEAS5 forecast for increasingly longer lead times to highlight the predictability of anomalous conditions. Months outside the traditional fire season are masked out.They are months with mean FWI lower then a third of the year maximum. The ENSO index helps identifying years of strong positive and negative anomalies with established El Niño or La Niña conditions. These years corresponds to period of high predictability when anomalous conditions could be predicted up to 7 months before.

The establishment of a positive or negative ENSO are usually monitored using a Multivariate index (MVI) obtained by extracting the leading combined Empirical Orthogonal Function (EOF) of five different variables over the tropical Pacific basin (30S–30 N and 100E–70 W). During strong positive and negative ENSO seasonal prediction of fire weather is enhanced up to 7 ahead (Fig. 4) as a results of the enhanced predictability of these large scale patterns at the seasonal time scale^44^. Efforts to mitigate the impact of fires during ENSO events in Indonesia could therefore benefit from an early warning system at this time scale as they could be issued with sufficient advance time. This could help enforcing land management practices, implement fire prevention and suppression measures, and raise awareness about the environmental and health hazards associated with burning^45^.

### Indian Ocean Dipole (IOD)

**Fig. 5:**
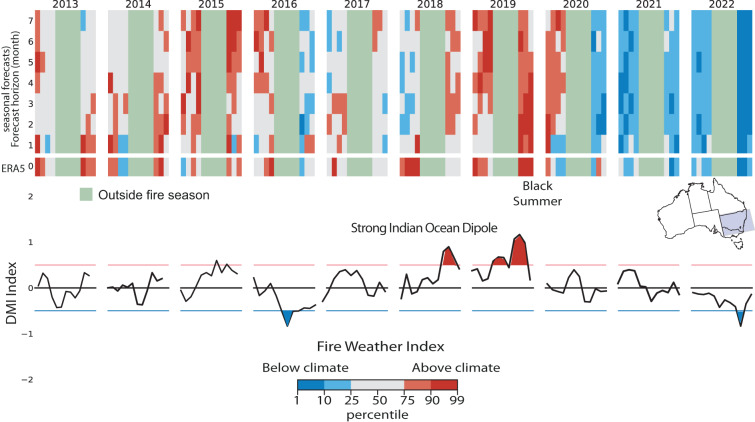
Prediction of monthly fire danger anomalous conditions between 2013 and 2022 over New Welsh in Australia. Months are classified as above or below the 1981–2022 mean climate using percentiles. Observed anomalies from ERA5 are compared to the forecast for increasingly longer lead times to highlight the predictability of anomalous fire weather conditions. Months outside the traditional fire season are masked out.They are defined as months with mean FWI lower then a third of the maximum yearly value. The Dipole mode index (DMI) helps identifying years of with established Indian Ocean Dipole conditions. DMI > 0.5 were recorded during the 2019 Black summer when anomalous conditions could be predicted up to 7 months before.

A similar phenomenon is the Indian Ocean Dipole (IOD) that occurs in the Indian Ocean, characterized by the difference in sea surface temperatures (SST) between the western and eastern parts of the ocean. The IOD has been known to influence weather patterns in various regions, including southern and eastern parts of Australia. During positive IOD events, there is typically a reduction in rainfall in these regions, leading to drier-than-normal conditions. There is still debate if there is a direct influence between the IOD and the Australian fires as a clear signal is often hindered by changing land management practices, fuel availability, and human activities^46^. Figure 5 shows the FWI anomalies over South east Australia for the 2013–2022 period in relation to the occurrence of the Indian Ocean Dipole as measured by the Dipole mode Index (DMI). The DMI is defined as the difference between the SST anomalies of Western (10S-10N and 50E-70E) and Eastern (10S-0N and 90E-110E) Equatorial Indian Ocean regions.

A more intricate picture emerges from the Australian case as there is not as strong a correlation between the DMI and the intensities of the fire seasons in Southwest Australia. For instance, in 2013–2014 and 2018, anomalous values of FWI were evident, yet they occurred independently of any DMI anomalies. During these years, the predictive capacity of the seasonal forecast was limited to just 1 month ahead. However, a prediction of anomalous conditions could be made for the 2019-2020 fire season, 7 months in advance due to the concurrent strong Indian Ocean Dipole.

The 2019-2020 Australian bushfire season, often termed the “Black Summer,” was exceptionally devastating and prolonged, occurring from late 2019 to early 2020. These fires had a severe impact on various parts of Australia, resulting in widespread destruction, loss of human lives, and significant damage to wildlife and the environment. The Black Summer fires were marked by their unprecedented scale, intensity, and duration.

In the aftermath of the Black Summer fires, efforts were undertaken to assess the damage and implement measures aimed at preventing and mitigating future fire season impacts. The 7-month predictability window for this extreme event could prove relevant in instituting sustainable practices to guard against future fire disasters.

## Usage Notes

In this section, we provide a typical workflow to retrieve and explore seasonal data using Python Jupyter Notebooks that is made available trough this github account https://github.com/fdg10371/CDS-jn-seasonal. In order to replicate the work, users should ahead over to the CDS website (https://cds.climate.copernicus.eu/cdsapp#!/home) and register an account (https://cds.climate.copernicus.eu/user/register?destination=%2F%23!%2Fhome). Once an account is created, and the user logs in, the seasonal fire forecasts can be found by typing relevant keywords in the search box, e.g. ‘fire danger indices seasonal data’. The web page dedicated to the seasonal fire forecasts is divided into three tabs: the ‘overview’ tab shows a concise description of the data; the ‘download data’ tab contains a data request form; the ‘documentation’ tab contains in depth information about the dataset and originating systems.

## Data Availability

The fire indices have been generated using the open source GEFF modelling system v4.1(https://github.com/ecmwf-projects/geff). The code to reproduce the results of this manuscript is openly available on a public repository: https://github.com/fdg10371/Jupyter_notebooks.
